# Association between *TMPRSS2* rs2070788 polymorphism and COVID-19 severity: a case-control study in multiple cities of Iran

**DOI:** 10.3389/fmed.2024.1425916

**Published:** 2024-08-12

**Authors:** Arezoo Faridzadeh, Mahmoud Mahmoudi, Bahman Rahimlou, Mohammad Mehdi Naghizadeh, Tooba Ghazanfari

**Affiliations:** ^1^Immunology Research Center, Mashhad University of Medical Sciences, Mashhad, Iran; ^2^Department of Immunology and Allergy, School of Medicine, Mashhad University of Medical Sciences, Mashhad, Iran; ^3^Immunoregulation Research Center, Shahed University, Tehran, Iran; ^4^Non-Communicable Diseases Research Center, Fasa University of Medical Science, Fasa, Iran; ^5^Department of Immunology, Shahed University, Tehran, Iran

**Keywords:** coronavirus disease 2019, *TMPRSS2*, SNP, polymorphism, rs2070788

## Abstract

**Introduction:**

Host genetic variations have been identified as potential influencers of COVID-19 infection. This study aimed to examine the association between transmembrane serine protease type 2 (*TMPRSS2*) rs2070788 single nucleotide polymorphism (SNP) and the prognosis of COVID-19 in Iranian populations.

**Method:**

This case-control study was performed on 756 COVID-19 patients and 59 healthy individuals across Iran. Clinical data, blood samples, and the presence of the *TMPRSS2* rs2070788: G>A SNP were determined using T-ARMS-PCR. Additionally, serum levels of tumor necrosis factor α (TNF-α), C-reactive protein (CRP), interleukin-6 (IL-6), and IL-1β were evaluated in the collected blood samples.

**Results:**

No significant association was found between the genotypes and allele frequencies of *TMPRSS2* rs2070788 SNP and susceptibility to or mortality from COVID-19 infection. However, we observed a substantial increase in IL-6 and CRP levels associated with the severity of COVID-19, while no such trend was observed for IL-1β and TNF-α. This study showed a considerable rise in TNF-α and IL-1β serum levels exclusively in COVID-19 patients with TT rs2070788 *TMPRSS2* SNP genotype compared to healthy controls.

**Conclusion:**

In this study conducted across multiple cities in Iran, no significant association was found between the *TMPRSS2* rs2070788 SNP genotypes and COVID-19 severity or mortality.

## 1 Introduction

The global impact of coronavirus disease 2019 (COVID-19), triggered by severe acute respiratory syndrome coronavirus 2 (SARS-CoV-2), has resulted in a considerable societal burden, encompassing elevated mortality and morbidity rates along with substantial economic expenditures (1). The World Health Organization estimates that this virus has infected 771 million individuals worldwide and that it has caused 6.9 million deaths.^1^

The clinical manifestations in individuals with COVID-19 span from being asymptomatic or experiencing mild flu-like symptoms to developing pneumonia, experiencing multi-organ failure, or succumbing to mortality (2, 3). Age, sex, underlying diseases, viral variants, and host genetic variations are recognized elements that may play a role in the prognosis of COVID-19 (4, 5). Hence, there is supportive evidence indicating that genetic diversity could potentially affect both the susceptibility to and the clinical consequences of SARS-CoV-2 infection (6–8).

The transmembrane serine protease type 2 (*TMPRSS2*) protein plays a vital role in the priming of viral spike proteins in coronavirus infections (9, 10). *TMPRSS2* is expressed in different organs, containing the gastrointestinal tract, heart, kidney, lung, and respiratory system, suggesting that their extensive distribution may enhance susceptibility to SARS-CoV-2 infection (11, 12).

According to the HUGO Gene Nomenclature Committee (HGNC), the *TMPRSS2* rs2070788: G˃A single nucleotide polymorphism (SNP) is situated on chromosome 21q22.3 within an intronic region. Studies have shown that this polymorphism influences *TMPRSS2* gene (PRSS10) expression (13–15). Several *in silico* studies investigated the potential role of this SNP in the prognosis of COVID-19 using genomic databases (16). A German case-control study demonstrated no association between *TMPRSS2* rs2070788 and the severity of COVID-19 (17).

By examining the importance of the *TMPRSS2* gene in the SARS-CoV-2 infection process, the incidence, and severity of COVID-19 may be directly related to the increased expression of the *TMPRSS2* gene, potentially leading to diverse consequences for disease susceptibility in different communities. Pandey et al. identified a significant positive correlation between the rs2070788 SNP (G allele) and the case fatality rate (CFR) in the Indian population. Also observed was the relationship between rs2070788(G) allele and *TMPRSS2* expression in the lungs (18). Martínez-Gómez et al. demonstrated an association between rs2070788 and the severity of COVID-19 in various Mexican populations (19). Mesquita et al. reported no significant association between the genotype distribution of rs2070788 and patient outcomes in a Brazilian population (20).

Novel approaches targeting the proteolytic action of *TMPRSS2* in viral pathogenesis and its potential blockade have been suggested as promising avenues to decrease mortality associated with SARS-CoV-2 infection (21, 22).

Consequently, this information may prove valuable in elucidating the significance of the *TMPRSS2* rs2070788 polymorphism concerning susceptibility and severity of COVID-19. Utilizing this polymorphism as a promising biomarker holds the potential to predict populations at risk. The extent to which host-specific genetic variations contribute to the severity of the cytokine storm and thereby influence the outcomes of COVID-19 is poorly understood and requires further investigation (23). In response to these findings, we initiated the first Iranian study to investigate the possible correlation between the *TMPRSS2* rs2070788 polymorphism and COVID-19 outcomes, with a particular focus on inflammatory markers.

## 2 Method

### 2.1 Study participants

This study involved 756 individuals diagnosed with COVID-19 and 59 healthy control subjects. These participants were recruited from hospitals in different major cities across Iran between 2020 and 2021. All participants in the study had not received the vaccine for SARS-CoV-2. Confirmed cases of COVID-19 were determined through either real-time reverse transcription-polymerase chain reaction (RT-PCR) testing or chest CT scan results. The COVID-19 cases severity was classified into four groups—mild, moderate, severe, and critical—according to the classification established by the World Health Organization (WHO) (24). The research protocol received approval from the National Institute for Medical Research Development under the reference number IR.NIMAD.REC.1399.041.

### 2.2 Data collection

We gathered all medical histories and relevant personal information of the participants, including age, gender, underlying health conditions, and smoking habits, by administering a patient checklist. Before collecting blood samples, informed consent was obtained from all participants themselves or their respective family members. At the onset of hospitalization, 5 mL blood samples were taken from both patients and healthy control individuals. These samples were collected using tubes containing clot activator and tubes containing ethylenediaminetetraacetic acid (EDTA).

### 2.3 C-reactive protein and pro-inflammatory cytokines assessment

Serum samples were subjected to an automated immunoassay (IMMULITE 2000; Siemens Healthcare Diagnostics, United Kingdom) to quantify the levels of interleukin-6 (IL-6). IL-1β, tumor necrosis factor (TNF)-α, and C-reactive protein (CRP) levels were determined in serum samples using the 7,180 clinical analyzers (Hitachi, Japan). The analysis of TNF-α, IL-1β, and CRP was conducted using the R&D Systems biotech brand kit and ACE BIOLIS (Genbio, Ireland).

### 2.4 Genotyping of *TMPRSS2* rs2070788 polymorphism

The DNA from the buffy coat samples of all participants was extracted utilizing a spin column kit (GenAll Exgene Cell SV mini kit, GenAll Biotechnology, South Korea).

The *TMPRSS2* rs2070788 polymorphism was assessed using Tetra-primer amplification refractory mutation system PCR (T-ARMS-PCR). The PRIMER1 software, available at http://primer1.soton.ac.uk/primer1.html, was employed to design T-ARMS-PCR primer pairs. The specific primers used are shown in Table 1.

**Table 1:** Primers sequences of *TMPRSS2* rs2070788 polymorphism.

Primers	Sequences
*TMPRSS2* rs2070788	Forward inner prime: 5′–TTGTTTATATCCTTCTCAAACGC-3′
	Reverse inner primer: 5′–TGTCTGTATGGCCTAGCCA-3′
	Forward outer primer: 5′–ACTCATTGTGAGTTTAGAGCTGC-3′
	Reverse outer primer: 5′–GATATTCCCTTCATCTTGGATTC-3′

**Figure 1:**
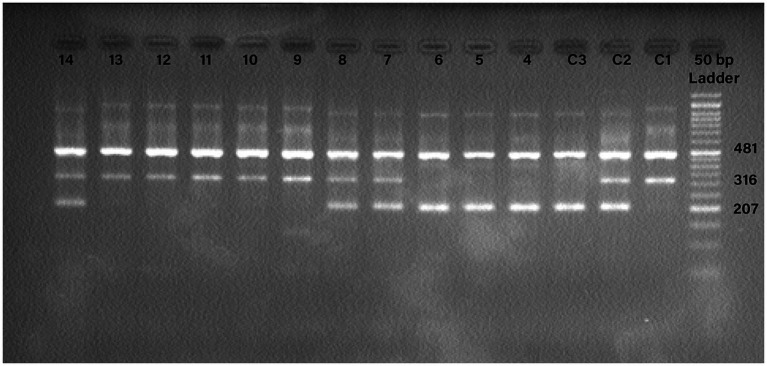
Detection of the PCR products for TMPRSS2 rs2070788 polymorphism. Internal control band 481 base pair (bp) is found in all wells, with band 207 bp representing allele C and band 316 bp representing allele T. C1: control TT; C2: control CT; C3: control CC; 4, 5, 6: CC; 7, 8, 14: CT; 9, 10, 11, 12, 13: TT.

The T-ARMS-PCR procedure was conducted using a total volume of 20 μL. This volume comprised 10 μL of TEMPase Hot Start 2X Master Mix (Ampliqon, Denmark), 1 μL of outer forward primer at a concentration of 10 pmol, 1 μL of outer reverse primer at a concentration of 10 pmol, 2 μL of inner forward primer at a concentration of 10 pmol, 2 μL of inner reverse primer at a concentration of 10 pmol, 2 μL of genomic DNA (ranging in concentration from 10 to 100 ng/μL), and 2 μL of Distillation Water. The PCR protocol commenced with an initial denaturation step at 95°C, lasting for 15 min. Subsequently, a total of 38 cycles were performed, consisting of denaturation at 95°C for 30 s, annealing at 58°C for 30 s, and extension at 72°C for 60 s. The procedure was finalized with a concluding extension step at 72°C for 5 min. To analyze the PCR outcomes, electrophoresis was conducted on a 2% agarose gel (Figure 1).

**Figure 2:**
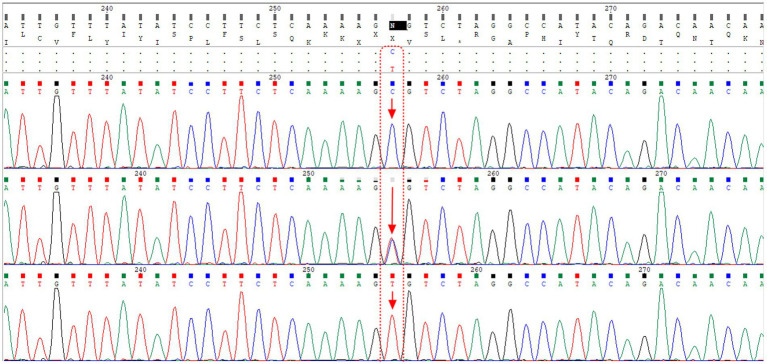
The alignment of sequencing results for the rs2070788 SNP, located in the TMPRSS2 gene, reveals that the first, second, and third rows correspond to samples with CC genotype, CT genotype, and TT genotype, respectively. Notably, the second row shows the presence of both peaks for both alleles.

To validate the T-ARMS-PCR for SNP rs2070788, approximately 10% of the samples underwent direct Sanger sequencing. Sanger sequencing was carried out on a subset of the samples using PCR primers. The alignment of the sequencing results for the rs2070788 SNP is presented in Figure 2.

### 2.5 Statistical analysis

The mean ± standard deviation was used to present the numerical variables in each group, and a Mann–Whitney test was conducted to compare the continuous data. The genotype frequencies were presented for each group as both the number and percentages (*n* %) and were subjected to evaluation through the Chi-square test. The Hardy–Weinberg equilibrium (HWE) was assessed using the Chi-square test. To examine the relationship between the *TMPRSS2* rs2070788 polymorphism and susceptibility and severity of SARS-CoV-2 infection, multinomial or binary logistic regressions were performed. Odds ratios (ORs), both adjusted and unadjusted, along with their corresponding 95% confidence intervals (CIs), were computed. The analysis included adjustments for age, sex, diabetes mellitus (DM), cardiovascular disease (CVD), hypertension (HTN), renal disease (RD), and cigarette smoking. Statistical significance was ascertained based on a *p*-value below 0.05.

## 3 Results

### 3.1 Demographic characteristics

This study involved 756 patients with COVID-19 and 59 healthy controls. Patients were stratified into groups based on the severity of the disease (25). The frequencies of comorbidities such as DM, CVD, HTN, RD, and cigarette smoking can be found in Supplementary Table S1 and Table 2. Also, DM (*p* = 0.026), HTN (*p* = 0.019), RD (*p* = 0.005), and CVD (*p* = 0.021) were significantly correlated with COVID-19 mortality. No significant difference in gender was observed among the groups. The mean age showed a correlation with higher disease severity and mortality rates in COVID-19. Specifically, the mean age of various groups including healthy controls, outpatients, inpatients, ICU-admitted patients, intubated patients, and expired patients was as follows: 39.4 ± 12.1, 42.7 ± 13.5, 57.4 ± 16.7, 61.9 ± 16.6, 61.3 ± 14.6, and 65.5 ± 13.7 years, respectively. A CONSORT diagram was incorporated into the study analysis to present an overview of the entire population count process (see Supplementary Figure S1).

**Table 2:** Demographic characteristics of the study participants.

	Control (*n* = 59)	COVID-19				
		Outpatient (*n* = 250)	Inpatient (*n* = 506)	Admitted to ICU (*n* = 129)	Intubated (*n* = 84)	Expired (*n* = 89)
Gender (Female ratio)	33 (55.9%)	107 (42.8%)	204 (40.3%)	52 (40.3%)	31 (36.9%)	30 (33.7%)
Cigarette smoking	4 (6.8%)	23 (9.2%)	93 (18.4%)	22 (17.1%)	14 (16.7%)	12 (13.5%)
HTN	2 (3.4%)	36 (14.4%)	189 (37.4%)	56 (43.4%)	31 (36.9%)	36 (40.4%)
DM	1 (1.7%)	28 (11.2%)	155 (30.6%)	49 (38%)	31 (36.9%)	30 (33.7%)
CVD	0 (0%)	12 (4.8%)	118 (23.3%)	37 (28.7%)	20 (23.8%)	23 (25.8%)
RD	0 (0%)	11 (4.4%)	44 (8.7%)	18 (14%)	12 (14.3%)	13 (14.6%)

*N*, number; %, frequency; COVID-19, coronavirus disease 2019; HTN, hypertension; DM, diabetes mellitus; CVD, cardiovascular disease; RD, renal disease.

### 3.2 *TMPRSS2* rs2070788 genotypes

The distribution of genotype frequencies for the *TMPRSS2* rs2070788 polymorphism in both the patient and control groups demonstrated conformity to the Hardy–Weinberg equilibrium, as confirmed through Chi-square analysis (see Supplementary Table S2).

Statistical comparisons of *TMPRSS2* genotypes/alleles between controls and patients (*p*-value).

No significant association was observed between the frequencies of different *TMPRSS2* rs2070788 genotypes/alleles and the presence of comorbidities. The corresponding statistical information can be found in Supplementary Table S3.

### 3.3 Susceptibility to COVID-19 infection

No significant correlation was observed between the genotypes/allele frequencies of the *TMPRSS2* rs2070788 SNP and the susceptibility to COVID-19 infection. Following adjustment for covariates including sex, age, DM, CVD, HTN, RD, and cigarette smoking, logistic regression analysis was conducted to compare 756 COVID-19 patients with 59 healthy controls. The findings indicated that there was no notable correlation between *TMPRSS2* rs2070788 SNP genotypes and susceptibility to COVID-19 infection (Table 3).

**Table 3:** Association of *TMPRSS2* genotypes/alleles distribution with susceptibility to COVID-19, adjusted by age, sex, cigarette smoking, diabetes mellitus, HTN, CVD, and RD.

Genotypes Alleles *N* (%)		Study group		Unadjusted		Adjusted	
		Control (*n* = 59)	COVID-19 (*n* = 756)	*p*-value	OR-95%CI (L-U)	*p*-value	OR-95%CI (L-U)
*TMPRSS2* rs2070788	CC	14 (23.7%)	195 (25.8%)				
	CT	29 (49.2%)	376 (49.7%)	0.882	0.855 (0.434–1.682)	0.468	0.771 (0.382–1.556)
	TT	16 (27.1%)	185 (24.5%)		0.766 (0.359–1.637)	0.336	0.678 (0.307–1.496)
	CC	14 (23.7%)	195 (25.8%)				
	TT + CT	45 (76.3%)	561 (74.2%)	0.726	0.823 (0.435–1.559)	0.368	0.738 (0.381–1.43)
	CC + CT	43 (72.9%)	571 (75.5%)				
	TT	16 (27.1%)	185 (24.5%)	0.650	0.852 (0.468–1.551)	0.500	0.805 (0.428–1.513)
	C	57 (48.3%)	766 (50.7%)				
	T	61 (51.7%)	746 (49.3%)	0.662			

The significant *p* values are in bold, *N* (%): number (percentage). *TMPRSS2*, Transmembrane Serine Protease 2; OR, odds ratio; CI, confidence interval; L, lower; U, upper; COVID-19, coronavirus disease 2019; HTN, Hypertension; DM, Diabetes mellitus; CVD, Cardiovascular disease; RD, renal disease.

Power = 0.978, effect size = 0.066 (small effect). Power and effect size were calculated for comparison of genotype (CC, CT, TT) between study groups.

### 3.4 Severity and mortality of COVID-19

The frequencies of different genotypes/alleles of *TMPRSS2* rs2070788 were not correlated with the severity and mortality of COVID-19 compared to different groups. After adjustment, binary logistic regression again showed no association (Tables 4, 5; Supplementary Tables S4–S6).

**Table 4:** Association *TMPRSS2* genotypes/alleles distribution with susceptibility to COVID-19, adjusted by age, sex, cigarette smoking, DM, HTN, CVD, and RD.

Genotypes Alleles *N* (%)		Study group		Unadjusted		Adjusted	
		Non-severe (*n* = 627)	Severe (*n* = 129)	*p*-value	OR-95%CI (L-U)	*p*-value	OR-95%CI (L-U)
*TMPRSS2* rs2070788	CC	163 (26.0%)	32 (24.8%)				
	CT	316 (50.4%)	60 (46.5%)	0.470	0.98 (0.613–1.567)	0.592	0.873 (0.533–1.432)
	TT	148 (23.6%)	37 (28.7%)		1.198 (0.706–2.033)	0.911	1.032 (0.592–1.8)
	CC	163 (26.0%)	32 (24.8%)				
	TT + CT	464 (74.0%)	97 (75.2%)	0.778	1.05 (0.677–1.63)	0.745	0.926 (0.583–1.47)
	CC + CT	479 (76.4%)	92 (71.3%)				
	TT	148 (23.6%)	37 (28.7%)	0.222	1.214 (0.789–1.868)	0.600	1.129 (0.718–1.775)
	C	642 (51.2%)	124 (48.1%)				
	T	612 (48.8%)	134 (51.9%)	0.359			

Non-Severe: Outpatients + Inpatients (not ICU). Severe: ICU + Intubated.

The significant *p* values are in bold, *N* (%): number (percentage). TMPRSS, Transmembrane Serine Protease 2; OR, odds ratio; CI, confidence interval; L, lower; U, upper; ICU, Intensive Care Unit; HTN, hypertension; DM, diabetes mellitus; CVD, cardiovascular disease; RD, renal disease.

Power = 0.990, effect size = 0.121 (small effect). Power and effect size were calculated for comparison of genotype (CC, CT, TT) between study groups.

**Table 5:** Association of *TMPRSS2* genotypes distribution with COVID-19 mortality, adjusted by age, sex, cigarette smoking, DM, HTN, CVD, and RD.

Genotypes Alleles *N* (%)		Mortality		Unadjusted		Adjusted	
		Survived (*n* = 667)	Expired (*n* = 89)	*p*-value	OR-95%CI (L-U)	*p*-value	OR-95%CI (L-U)
*TMPRSS2* rs2070788	CC	173 (25.9%)	22 (24.7%)				
	CT	337 (50.5%)	39 (43.8%)	0.250	0.895 (0.513–1.562)	0.511	0.82 (0.453–1.482)
	TT	157 (23.5%)	28 (31.5%)		1.354 (0.741–2.476)	0.662	1.154 (0.606–2.197)
	CC	173 (25.9%)	22 (24.7%)				
	TT + CT	494 (74.1%)	67 (75.3%)	0.805	1.042 (0.623–1.742)	0.800	0.932 (0.539–1.611)
	CC + CT	510 (76.5%)	61 (68.5%)				
	TT	157 (23.5%)	28 (31.5%)	0.102	1.455 (0.892–2.371)	0.304	1.314 (0.781–2.212)
	C	683 (51.2%)	83 (46.6%)				
	T	651 (48.8%)	95 (53.4%)	0.252			

The significant *p* values are in bold, *N* (%): number (percentage). *TMPRSS2*, Transmembrane Serine Protease 2, OR, odds ratio; CI, confidence interval; L, lower; U, upper; HTN, hypertension; DM, diabetes mellitus; CVD, cardiovascular disease; RD, renal disease.

Power = 0.999, effect size = 0.192 (small effect). Power and effect size were calculated for comparison of genotype (CC, CT, TT) between mortality.

### 3.5 Clinical laboratory data

#### 3.5.1 C-reactive protein

C-reactive protein (CRP) elevated along with the severity of COVID-19 in all genotypes., as shown in Supplementary Tables S7–S12. However, the mean ± SD serum levels of CRP in ICU-admitted and intubated patients only with TT + CT genotypes were shown to be significantly higher than those with TT + CT genotypes who were not admitted to the ICU or intubated (24.87 ± 20.88 vs. 19.44 ± 10.71, *p* = 0.001, 23.46 ± 8.05 vs. 20.26 ± 14.87, *p* = 0.005, respectively).

#### 3.5.2 Interleukin-6

**Figure 3:**
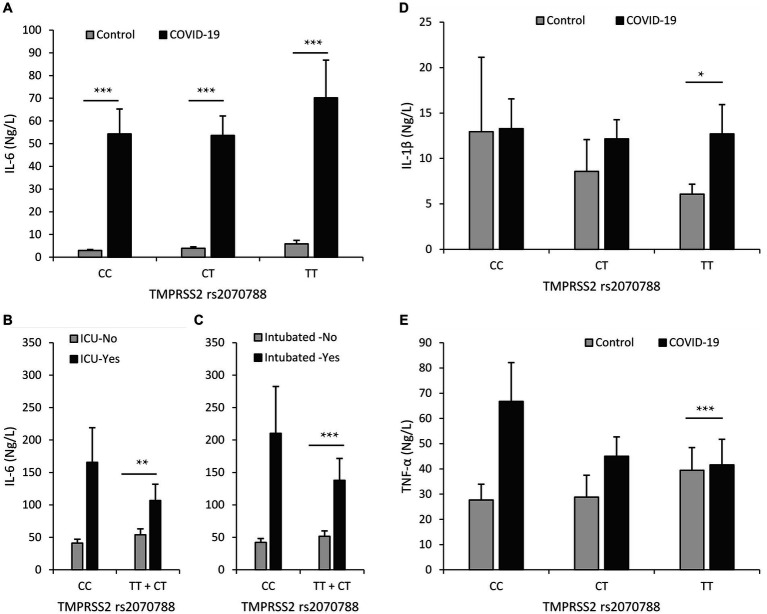
Relationship between serum levels of inflammatory cytokines and rs2070788 polymorphism genotypes. **(A)** IL-6 serum level in the COVID-19 group vs. the control group, **(B)** IL-6 serum level in the ICU admission group vs. ward admission, **(C)** IL-6 serum level in the intubated group vs. the not intubated group, **(D)** IL-1β serum level in the COVID-19 group vs. the control group, **(E)** TNF-α serum level in the COVID-19 group vs. the control group. *p < 0.05, **p < 0.01, ***p < 0.001, indicating statistically significant differences between groups.

Among all individuals carrying *TMPRSS2* rs2070788 genotypes, the IL-6 levels exhibited a significant elevation within the COVID-19 group when contrasted with the control group. Additionally, it was noticeably higher in inpatients compared to outpatients, in intubated patients compared to those not intubated, and in those who expired compared to those who survived. However, in the group that was ICU admitted, it was notably higher only in the carriers of the heterozygous genotype compared to the group that was hospitalized in the department. However, among the group that was admitted to the ICU, it was remarkably higher only in carriers of the heterozygous genotype compared to the group that was hospitalized in the regular department (Supplementary Tables S13–18; Figure 3).

#### 3.5.3 Interleukin-1β

The level of Interleukin-1β (IL-1β) was elevated in COVID-19 patients compared to controls; however, this increase was determined to be significant only in carriers of the TT genotype. However, we observed that the level of IL-1β does not increase with the severity of COVID-19 disease (Supplementary Tables S19–S24; Figure 3).

#### 3.5.4 Tumor necrosis factor-α

In COVID-19 patients, the level of tumor necrosis factor-α (TNF-α) was higher compared to controls. However, this increase was found to be significant only in individuals with the TT genotype. Nevertheless, our observations indicate that the level of TNF-α does not rise in correlation with the severity of COVID-19 disease (Supplementary Tables S25–S30; Figure 3).

## 4 Discussion

In this case-control study, the distribution of *TMPRSS2* rs2070788 genotypes was found to be consistent with the Hardy–Weinberg equilibrium, suggesting that the chosen samples reflected the broader population. This study is the first to examine the potential correlation between the genetic factor *TMPRSS2* rs2070788 SNP and COVID-19 severity in multiple predominant cities across Iran.

Following the initial evidence highlighting the significance of *TMPRSS2* in the entry of SARS-CoV-2 (11), numerous studies have emerged aiming to explore the link between genetic variations in *TMPRSS2* and the susceptibility to COVID-19 (26). These investigations have made use of genomic databases (13, 14, 27–30). Among the numerous single nucleotide polymorphisms (SNPs) present in the *TMPRSS2* gene, certain variants, such as rs2070788 and rs12329760, have displayed potential associations as they have been linked to alterations in *TMPRSS2* expression levels (13, 29, 31).

The first study to emphasize the significance of the *TMPRSS2* rs2070788 SNP in the physiological mechanisms underlying viral respiratory infections was conducted on a cohort of Asian patients infected with H1N1 influenza. This study revealed that individuals carrying the rs2070788 CC genotype had a risk of severe H1N1 influenza more than twofold higher than individuals with other genotypes (31). Additionally, a recent study conducted in the Netherlands, which included 188 adult patients admitted to the hospital, exhibited a protective effect associated with the rs2070788 AA genotype against the severity of COVID-19 (32).

Conversely, a case-control study conducted in Germany, which examined 239 patients diagnosed with COVID-19, did not identify any association between the *TMPRSS2* rs2070788 polymorphism and the severity of the disease (17). A cross-sectional study conducted in Spain did not find any association between *TMPRSS2* rs2070788 polymorphism and long-term COVID-19 symptoms (33).

In line with these findings, our results also showed no considerable correlation between the frequency of genotypes of the *TMPRSS2* rs2070788 polymorphism and the severity or mortality of COVID-19. Furthermore, across all *TMPRSS2* rs2070788 genotypes, there was an observed increase in IL-6 and CRP levels in conjunction with disease severity. Conversely, no significant associations were found between disease severity and TNF-α or IL-1β levels. Previous studies have also noted a significant increase in IL-6 and CRP levels in association with disease severity (6, 34). In our study, a significant increase in IL-1β and TNF-α serum levels was exclusively observed in carriers of the TT genotype of the rs2070788 *TMPRSS2* SNP in COVID-19 patients compared to healthy controls.

The precise functional mechanism through which the rs2070788 SNP, located in the noncoding region of the *TMPRSS2* gene, influences the outcome of COVID-19 remains uncertain and necessitates additional research. One potential explanation is that this polymorphism could impact the stability of *TMPRSS2* mRNA, including splicing processes, post-transcriptional regulation mediated by microRNAs, and the effectiveness of mRNA splicing. Factors such as the presence of silencing elements or enhancers within introns could potentially contribute to these effects.

## 5 Limitation

One of the study’s limitations pertained to insufficient blood sample volumes obtained from some patients, which hindered laboratory tests, including measurements of CRP, IL-1β, TNF-α, and IL-6 serum levels. Additionally, although the assessment of *TMPRSS2* expression could have offered additional insights into forecasting the outcome of COVID-19, there were no remaining blood samples available to conduct these specific tests. Moreover, the control group had a relatively small sample size.

## 6 Conclusion

In conclusion, this case-control study examined the association between the *TMPRSS2* rs2070788 SNP and COVID-19 severity in multiple cities across Iran. The findings revealed no significant correlation between the frequency of genotypes of this polymorphism and the severity or mortality of COVID-19. However, an increase in IL-6 and CRP levels was observed with disease severity across all genotypes, while no significant associations were found with TNF-α or IL-1β levels. The functional mechanism by which the rs2070788 SNP affects COVID-19 outcomes remains unclear and requires further investigation. The study focused on the need for continued research to fully understand the role of *TMPRSS2* in COVID-19 pathophysiology and its potential implications for disease management and treatment strategies. Further research involving diverse ethnicities and larger sample sizes is essential to corroborate our findings. Genetic studies in the future can be influential in personalized medicine.

## Data Availability

The datasets presented in this study can be found in online repositories. The names of the repository/repositories and accession number(s) can be found in the article/Supplementary material.

## Footnotes

^1^https://covid19.who.int/ accessed on 4 October 2023.
