# Supplementary material for: Association between TMPRSS2 rs2070788 polymorphism and COVID-19 severity: a case-control study in multiple cities of Iran
Source: Front Med (Lausanne). 2024 Aug 12;11:1425916. doi: 10.3389/fmed.2024.1425916 (PMC11345270; doi:10.3389/fmed.2024.1425916)
Supplement: Supplementary file 1 [file Data_Sheet_1.docx]

Study Participants (n=815)

## *TMPRSS2* rs2070788 genotypes

Healthy Control (n=59)

COVID-19 (n=756)

- Outpatient (n=250)
- Inpatient (n=506)
- Admitted to ICU (n=129)
- Intubated (n=84)
- Expired (n=89)

Excluded from analysed: Insufficient blood sample volumes

## Clinical laboratory data

**C-reactive protein**

- COVID-19 (n=699)
- Control (n=59)

**Interleukin-6**

- COVID-19 (n=544)
- Control (n=48)

**Tumor Necrosis Factor-α**

- COVID-19 (n=627)
- Control (n=58)

**Interleukin-1β**

- COVID-19 (n=627)
- Control (n=59)

Figure S1: Study CONSORT diagram
