# Supplementary material for: Association between TMPRSS2 rs2070788 polymorphism and COVID-19 severity: a case-control study in multiple cities of Iran
Source: Front Med (Lausanne). 2024 Aug 12;11:1425916. doi: 10.3389/fmed.2024.1425916 (PMC11345270; doi:10.3389/fmed.2024.1425916)
Supplement: Supplementary file 2 [file Table_1.DOCX]

Supplementary Table S1: Association between Demographic Characteristics and Severity of COVID-19.

| Variables | | Control  (n=59) | COVID-19 (n=756) | | | Inpatient (n=506) | | | | | | COVID-19 (n=756) | | |
| --- | --- | --- | --- | --- | --- | --- | --- | --- | --- | --- | --- | --- | --- | --- |
|  |  |  |  |  |  | ICU | | | Intubation | | |  |  |  |
|  |  |  | Outpatient  (n=250) | Inpatient  (n=506) | P-value | No  (n=377) | Yes  (n=129) | P-value | No  (n=423) | Yes  (n=83) | P-value | Survived  (n = 667) | Expired  (n=89) | P-value |
| Sex | Female | 33 (55.9%) | 107 (42.8%) | 204 (40.3%) |  | 152 (40.3%) | 52 (40.3%) |  | 174 (41.1%) | 30 (36.1%) |  | 281 (42.1%) | 30 (33.7%) |  |
|  | Male | 26 (44.1%) | 143 (57.2%) | 302 (59.7%) | 0.069 | 225 (59.7%) | 77 (59.7%) | 0.999 | 249 (58.9%) | 53 (63.9%) | 0.397 | 386 (57.9%) | 59 (66.3%) | 0.129 |
| Cigarette Smoking | No | 55 (93.2%) | 227 (90.8%) | 413 (81.6%) |  | 306 (81.2%) | 107 (82.9%) |  | 344 (81.3%) | 69 (83.1%) |  | 563 (84.4%) | 77 (86.5%) |  |
|  | Yes | 4 (6.8%) | 23 (9.2%) | 93 (18.4%) | **0.001** | 71 (18.8%) | 22 (17.1%) | 0.653 | 79 (18.7%) | 14 (16.9%) | 0.697 | 104 (15.6%) | 12 (13.5%) | 0.604 |
| HTN | No | 57 (96.6%) | 214 (85.6%) | 317 (62.6%) |  | 244 (64.7%) | 73 (56.6%) |  | 265 (62.6%) | 52 (62.7%) |  | 478 (71.7%) | 53 (59.6%) |  |
|  | Yes | 2 (3.4%) | 36 (14.4%) | 189 (37.4%) | **<0.001** | 133 (35.3%) | 56 (43.4%) | 0.099 | 158 (37.4%) | 31 (37.3%) | 0.999 | 189 (28.3%) | 36 (40.4%) | **0.019** |
| DM | No | 58 (98.3%) | 222 (88.8%) | 351 (69.4%) |  | 271 (71.9%) | 80 (62.0%) |  | 299 (70.7%) | 52 (62.7%) |  | 514 (77.1%) | 59 (66.3%) |  |
|  | Yes | 1 (1.7%) | 28 (11.2%) | 155 (30.6%) | **<0.001** | 106 (28.1%) | 49 (38.0%) | **0.036** | 124 (29.3%) | 31 (37.3%) | 0.147 | 153 (22.9%) | 30 (33.7%) | **0.026** |
| CVD | No | 59 (100.0%) | 238 (95.2%) | 388 (76.7%) |  | 296 (78.5%) | 92 (71.3%) |  | 325 (76.8%) | 63 (75.9%) |  | 560 (84.0%) | 66 (74.2%) |  |
|  | Yes | 0 (0.0%) | 12 (4.8%) | 118 (23.3%) | **<0.001** | 81 (21.5%) | 37 (28.7%) | 0.095 | 98 (23.2%) | 20 (24.1%) | 0.855 | 107 (16.0%) | 23 (25.8%) | **0.021** |
| RD | No | 59 (100.0%) | 239 (95.6%) | 462 (91.3%) |  | 351 (93.1%) | 111 (86.0%) |  | 391 (92.4%) | 71 (85.5%) |  | 625 (93.7%) | 76 (85.4%) |  |
|  | Yes | 0 (0.0%) | 11 (4.4%) | 44 (8.7%) | **0.009** | 26 (6.9%) | 18 (14.0%) | **0.014** | 32 (7.6%) | 12 (14.5%) | **0.042** | 42 (6.3%) | 13 (14.6%) | **0.005** |

ICU: Intensive care unit; HTN: Hypertension; DM: Diabetes mellitus; RD: renal disease; CVD: Cardiovascular disease; COVID-19: Coronavirus disease 2019.

Supplementary Table S2: Hardy-Weinberg Equilibrium.

|  | | **Control** | | **COVID-19** | | **Outpatient** | | **Inpatient** | |
| --- | --- | --- | --- | --- | --- | --- | --- | --- | --- |
|  |  | Observed | Expected | Observed | Expected | Observed | Expected | Observed | Expected |
| **TMPRSS2**  **rs2070788** | CC | 14 | 13.8 | 195 | 194.0 | 64 | 61.5 | 131 | 132.6 |
|  | CT | 29 | 29.5 | 376 | 377.9 | 120 | 125.0 | 256 | 252.9 |
|  | TT | 16 | 15.8 | 185 | 184.0 | 66 | 63.5 | 119 | 120.6 |
|  | P | 0.483 | | 0.507 | | 0.496 | | 0.512 | |
|  | q | 0.517 | | 0.493 | | 0.504 | | 0.488 | |
|  | x2 | 0.015 | | 0.020 | | 0.399 | | 0.078 | |
|  | P-value | 0.903 | | 0.888 | | 0.528 | | 0.780 | |

TMPRSS2, Transmembrane Serine Protease 2.

Supplementary Table S3: Association Genotype Frequencies of the TMPRSS2 Polymorphism with HTN, DM, CVD, and RD of Individuals in COVID-19 Patients.

|  |  | rs2070788 Polymorphism | | |  | Dominant | |  | Recessive | |  |
| --- | --- | --- | --- | --- | --- | --- | --- | --- | --- | --- | --- |
|  |  | CC | CT | TT | P-value | CC | TT + CT | P-value | CC + CT | TT | P-value |
| HTN | No | 140 (26.4%) | 263 (49.5%) | 128 (24.1%) |  | 140 (26.4%) | 391 (73.6%) |  | 403 (75.9%) | 128 (24.1%) |  |
|  | Yes | 55 (24.4%) | 113 (50.2%) | 57 (25.3%) | 0.844 | 55 (24.4%) | 170 (75.6%) | 0.581 | 168 (74.7%) | 57 (25.3%) | 0.72 |
| DM | No | 156 (27.2%) | 283 (49.4%) | 134 (23.4%) |  | 156 (27.2%) | 417 (72.8%) |  | 439 (76.6%) | 134 (23.4%) |  |
|  | Yes | 39 (21.3%) | 93 (50.8%) | 51 (27.9%) | 0.215 | 39 (21.3%) | 144 (78.7%) | 0.111 | 132 (72.1%) | 51 (27.9%) | 0.219 |
| CVD | No | 168 (26.8%) | 302 (48.2%) | 156 (24.9%) |  | 168 (26.8%) | 458 (73.2%) |  | 470 (75.1%) | 156 (24.9%) |  |
|  | Yes | 27 (20.8%) | 74 (56.9%) | 29 (22.3%) | 0.177 | 27 (20.8%) | 103 (79.2%) | 0.15 | 101 (77.7%) | 29 (22.3%) | 0.528 |
| RD | No | 182 (26%) | 345 (49.2%) | 174 (24.8%) |  | 182 (26%) | 519 (74%) |  | 527 (75.2%) | 174 (24.8%) |  |
|  | Yes | 13 (23.6%) | 31 (56.4%) | 11 (20%) | 0.572 | 13 (23.6%) | 42 (76.4%) | 0.704 | 44 (80%) | 11 (20%) | 0.423 |
| Sex | Female | 69 (22.2%) | 165 (53.1%) | 77 (24.8%) |  | 69 (22.2%) | 242 (77.8%) |  | 234 (75.2%) | 77 (24.8%) |  |
|  | Male | 126 (28.3%) | 211 (47.4%) | 108 (24.3%) | 0.146 | 126 (28.3%) | 319 (71.7%) | 0.058 | 337 (75.7%) | 108 (24.3%) | 0.878 |
| Cigarette Smoking | No | 165 (25.8%) | 316 (49.4%) | 159 (24.8%) |  | 165 (25.8%) | 475 (74.2%) |  | 481 (75.2%) | 159 (24.8%) |  |
|  | Yes | 30 (25.9%) | 60 (51.7%) | 26 (22.4%) | 0.841 | 30 (25.9%) | 86 (74.1%) | 0.958 | 90 (77.6%) | 26 (22.4%) | 0.575 |
| Age | Mean (SD) | 50.7 (16.6) | 52.2 (17.4) | 54.8 (17.3) | 0.069 | 50.7 (16.6) | 53.1 (17.4) | 0.102 | 51.7 (17.1) | 54.8 (17.3) | 0.057 |

TMPRSS2: Transmembrane Serine Protease 2, HTN: Hypertension; DM: Diabetes mellitus; CVD: Cardiovascular disease; RD: renal disease.

.

Supplementary Table S4: Association TMPRSS2 Genotypes/ Alleles distribution with susceptibility to COVID-19, adjusted by age, sex, Cigarette smoking, DM, HTN, CVD, and RD.

| Genotypes  Alleles  N (%) | | **Study group** | | **Unadjusted** | | **Adjusted** | |  |
| --- | --- | --- | --- | --- | --- | --- | --- | --- |
|  |  | Outpatients  (n=250) | Inpatients  (n=506) | P-value | OR-95%CI- (L-U) | P-value | OR-95%CI- (L-U) | |
| TMPRSS2 rs2070788 | CC | 64 (25.6%) | 131 (25.9%) |  |  |  |  | |
|  | CT | 120 (48%) | 256 (50.6%) | 0.670 | 1.025 (0.708-1.485) | 0.650 | 0.908 (0.6-1.375) | |
|  | TT | 66 (26.4%) | 119 (23.5%) |  | 0.872 (0.57-1.335) | 0.081 | 0.651 (0.402-1.055) | |
|  | CC | 64 (25.6%) | 131 (25.9%) |  |  |  |  | |
|  | TT + CT | 186 (74.4%) | 375 (74.1%) | 0.932 | 0.971 (0.686-1.375) | 0.302 | 0.815 (0.552-1.202) | |
|  | CC + CT | 184 (73.6%) | 387 (76.5%) |  |  |  |  | |
|  | TT | 66 (26.4%) | 119 (23.5%) | 0.386 | 0.858 (0.605-1.217) | 0.073 | 0.693 (0.464-1.034) | |
|  | C | 248 (49.6%) | 518 (51.2%) |  |  |  |  | |
|  | T | 252 (50.4%) | 494 (48.8%) | 0.562 |  |  |  | |

TMPRSS2, Transmembrane Serine Protease 2; HTN, Hypertension; DM, Diabetes mellitus; RD, renal disease; CVD, Cardiovascular disease; COVID-19, Coronavirus disease 2019.

Supplementary Table S5: Association of TMRSS2 Genotypes/ Alleles Distribution with COVID-19 Severity, Adjusted by Age, Sex, Cigarette Smoking, DM, HTN, CVD, and RD.

| Genotypes  Alleles  N (%) | | **Inpatients (ICU Admitted)** | | **Unadjusted** | | **Adjusted** | | |
| --- | --- | --- | --- | --- | --- | --- | --- | --- |
|  |  | No  (n=377) | Yes  (n=129) | P-value | OR-95%CI- (L-U) | P-value | OR-95%CI- (L-U) |  |
| TMPRSS2 rs2070788 | CC | 99 (26.3%) | 32 (24.8%) |  |  |  |  |  |
|  | CT | 196 (52%) | 60 (46.5%) | 0.271 | 0.967 (0.59-1.586) | 0.734 | 0.916 (0.552-1.521) |  |
|  | TT | 82 (21.8%) | 37 (28.7%) |  | 1.31 (0.746-2.3) | 0.533 | 1.202 (0.674-2.145) |  |
|  | CC | 99 (26.3%) | 32 (24.8%) |  |  |  |  |  |
|  | TT + CT | 278 (73.7%) | 97 (75.2%) | 0.745 | 1.071 (0.674-1.701) | 0.990 | 1.003 (0.623-1.614) |  |
|  | CC + CT | 295 (78.2%) | 92 (71.3%) |  |  |  |  |  |
|  | TT | 82 (21.8%) | 37 (28.7%) | 0.109 | 1.339 (0.844-2.122) | 0.316 | 1.274 (0.794-2.045) |  |
|  | C | 394 (52.3%) | 124 (48.1%) |  |  |  |  |  |
|  | T | 360 (47.7%) | 134 (51.9%) | 0.245 |  |  |  |  |

The significant P values are in bold, n (%): number (percentage). Abbreviations: TMPRSS, Transmembrane Serine Protease 2; I, insertion; D, deletion; OR, odds ratio; CI, confidence interval; L, lower; U, upper; ICU, Intensive Care Unit; HTN, hypertension; DM, diabetes mellitus; CVD, cardiovascular disease; RD, renal disease.

Supplementary Table S6: Association of TMPRSS2 Genotypes Distribution with Intubation of COVID-19 Patients, Adjusted by Age, Sex, Cigarette Smoking, DM, HTN, CVD, and RD.

| Genotypes  Alleles  N (%) | | **Inpatients (Intubation)** | | **Unadjusted** | | **Adjusted** | | |
| --- | --- | --- | --- | --- | --- | --- | --- | --- |
|  |  | No  (n=422) | Yes  (n=84) | P-value | OR-95%CI- (L-U) | P-value | OR-95%CI- (L-U) |  |
| TMPRSS2 rs2070788 | CC | 111 (26.3%) | 20 (23.8%) |  |  |  |  |  |
|  | CT | 216 (51.2%) | 40 (47.6%) | 0.487 | 1.048 (0.584-1.881) | 0.926 | 1.029 (0.567-1.866) |  |
|  | TT | 95 (22.5%) | 24 (28.6%) |  | 1.348 (0.697-2.608) | 0.515 | 1.251 (0.637-2.456) |  |
|  | CC | 111 (26.3%) | 20 (23.8%) |  |  |  |  |  |
|  | TT + CT | 311 (73.7%) | 64 (76.2%) | 0.634 | 1.141 (0.659-1.975) | 0.741 | 1.099 (0.628-1.923) |  |
|  | CC + CT | 327 (77.5%) | 60 (71.4%) |  |  |  |  |  |
|  | TT | 95 (22.5%) | 24 (28.6%) | 0.232 | 1.307 (0.767-2.227) | 0.461 | 1.228 (0.712-2.117) |  |
|  | C | 438 (51.9%) | 80 (47.6%) |  |  |  |  |  |
|  | T | 406 (48.1%) | 88 (52.4%) | 0.311 |  |  |  |  |

The significant P values are in bold, N (%): number (percentage). Abbreviations: TMPRSS2, Transmembrane Serine Protease 2, I, insertion; D, deletion; OR, odds ratio; CI, confidence interval; L, lower; U, upper; HTN, hypertension; DM, diabetes mellitus; CVD, cardiovascular disease; RD, renal disease.

Supplementary Table S7: Association TMPRSS2 genotypes distribution with serum CRP level.

| Genotypes  Alleles  N (%) | | CRP | | | | | | | | | | |
| --- | --- | --- | --- | --- | --- | --- | --- | --- | --- | --- | --- | --- |
|  |  | Control | | | | | COVID-19 | | | | P-value |  |
|  |  | N | Mean | SD | Median | N | | Mean | SD | Median |  |  |
| TMPRSS2 rs2070788 | CC | 14 | 3.9 | 1.69 | 4.75 | 180 | | 14.69 | 10.64 | 15.25 | 0.002 |  |
|  | CT | 29 | 3.23 | 1.55 | 3.3 | 349 | | 15.67 | 11.94 | 17.1 | 0.000 |  |
|  | TT | 16 | 2.72 | 1.33 | 2.3 | 170 | | 15.61 | 17.91 | 14.85 | 0.000 |  |
|  | P-value |  | 0.287 |  |  |  | | 0.634 |  |  |  |  |
|  | CC | 14 | 3.9 | 1.69 | 4.75 | 180 | | 14.69 | 10.64 | 15.25 | 0.002 |  |
|  | TT + CT | 45 | 3.05 | 1.48 | 2.4 | 519 | | 15.65 | 14.15 | 16.4 | 0.000 |  |
|  | P-value |  | 0.141 |  |  |  | | 0.518 |  |  |  |  |
|  | CC + CT | 43 | 3.45 | 1.61 | 3.6 | 529 | | 15.34 | 11.51 | 16.5 | 0.000 |  |
|  | TT | 16 | 2.72 | 1.33 | 2.3 | 170 | | 15.61 | 17.91 | 14.85 | 0.000 |  |
|  | P-value |  | 0.298 |  |  |  | | 0.655 |  |  |  |  |
|  | Homo | 30 | 3.27 | 1.6 | 2.4 | 350 | | 15.14 | 14.61 | 15.2 | 0.000 |  |
|  | Hetero | 29 | 3.23 | 1.55 | 3.3 | 349 | | 15.67 | 11.94 | 17.1 | 0.000 |  |
|  | P-value |  | 0.744 |  |  |  | | 0.343 | 0.744 |  |  |  |

TMPRSS2: Transmembrane Serine Protease 2; CRP: C-Reactive Protein.

Supplementary Table S8: Association TMPRSS2 genotypes distribution with serum CRP level.

| Genotypes  Alleles  N (%) | | CRP | | | | | | | | | | |
| --- | --- | --- | --- | --- | --- | --- | --- | --- | --- | --- | --- | --- |
|  |  | Outpatient | | | | | Inpatient | | | | P-value |  |
|  |  | N | Mean | SD | Median | N | | Mean | SD | Median |  |  |
| TMPRSS2 rs2070788 | CC | 61 | 5.76 | 7.14 | 2.8 | 119 | | 19.27 | 9.13 | 21.1 | 0.000 |  |
|  | CT | 108 | 5.51 | 7.04 | 2.6 | 241 | | 20.22 | 10.83 | 21.8 | 0.000 |  |
|  | TT | 63 | 4.77 | 6.01 | 2.5 | 107 | | 22 | 19.47 | 21.6 | 0.000 |  |
|  | P-value |  | 0.637 |  |  |  | | 0.522 |  |  |  |  |
|  | CC | 61 | 5.76 | 7.14 | 2.8 | 119 | | 19.27 | 9.13 | 21.1 | 0.000 |  |
|  | TT + CT | 171 | 5.24 | 6.67 | 2.6 | 348 | | 20.77 | 14.06 | 21.75 | 0.000 |  |
|  | P-value |  | 0.372 |  |  |  | | 0.292 |  |  |  |  |
|  | CC + CT | 169 | 5.6 | 7.06 | 2.6 | 360 | | 19.9 | 10.3 | 21.65 | 0.000 |  |
|  | TT | 63 | 4.77 | 6.01 | 2.5 | 107 | | 22 | 19.47 | 21.6 | 0.000 |  |
|  | P-value |  | 0.979 |  |  |  | | 0.453 |  |  |  |  |
|  | Homo | 124 | 5.26 | 6.58 | 2.6 | 226 | | 20.56 | 14.98 | 21.3 | 0.000 |  |
|  | Hetero | 108 | 5.51 | 7.04 | 2.6 | 241 | | 20.22 | 10.83 | 21.8 | 0.000 |  |
|  | P-value |  | 0.444 |  |  |  | | 0.775 |  |  |  |  |

TMPRSS2: Transmembrane Serine Protease 2; CRP: C-Reactive Protein.

Supplementary Table S9: Association TMPRSS2 genotypes distribution with serum CRP level: Association TMPRSS2 genotypes distribution with serum CRP level.

Non-Severe: Outpatients + Inpatients (not ICU)

Severe: ICU _+_ Intubated

| Genotypes  Alleles  N (%) | | CRP | | | | | | | | | | |
| --- | --- | --- | --- | --- | --- | --- | --- | --- | --- | --- | --- | --- |
|  |  | Non-Severe | | | | | Severe | | | | P-value |  |
|  |  | N | Mean | SD | Median | N | | Mean | SD | Median |  |  |
| TMPRSS2 rs2070788 | CC | 152 | 13.49 | 10.61 | 11.95 | 28 | | 21.21 | 8.27 | 24 | 0.001 |  |
|  | CT | 294 | 14.34 | 12.03 | 13.55 | 55 | | 22.75 | 8.52 | 24.3 | 0.000 |  |
|  | TT | 140 | 12.8 | 10.7 | 9.2 | 30 | | 28.76 | 33.22 | 25.3 | 0.000 |  |
|  | P-value |  | 0.525 |  |  |  | | 0.450 |  |  |  |  |
|  | CC | 152 | 13.49 | 10.61 | 11.95 | 28 | | 21.21 | 8.27 | 24 | 0.001 |  |
|  | TT + CT | 434 | 13.85 | 11.62 | 12.35 | 85 | | 24.87 | 20.88 | 24.6 | 0.000 |  |
|  | P-value |  | 0.829 |  |  |  | | 0.260 |  |  |  |  |
|  | CC + CT | 446 | 14.05 | 11.56 | 12.8 | 83 | | 22.23 | 8.42 | 24.3 | 0.000 |  |
|  | TT | 140 | 12.8 | 10.7 | 9.2 | 30 | | 28.76 | 33.22 | 25.3 | 0.000 |  |
|  | P-value |  | 0.327 |  |  |  | | 0.356 |  |  |  |  |
|  | Homo | 292 | 13.16 | 10.64 | 11.45 | 58 | | 25.12 | 24.66 | 24.8 | 0.000 |  |
|  | Hetero | 294 | 14.34 | 12.03 | 13.55 | 55 | | 22.75 | 8.52 | 24.3 | 0.000 |  |
|  | P-value |  | 0.305 |  |  |  | | 0.874 |  |  |  |  |

TMPRSS2: Transmembrane Serine Protease 2; CRP: C-Reactive Protein.

Supplementary Table S10: Association TMPRSS2 genotypes distribution with serum CRP level.

| Genotypes  Alleles  N (%) | | CRP | | | | | | | | | | |
| --- | --- | --- | --- | --- | --- | --- | --- | --- | --- | --- | --- | --- |
|  |  | Inpatient ICU-No | | | | | Inpatient ICU-Yes | | | | P-value |  |
|  |  | N | Mean | SD | Median | N | | Mean | SD | Median |  |  |
| TMPRSS2 rs2070788 | CC | 91 | 18.67 | 9.35 | 20.5 | 28 | | 21.21 | 8.27 | 24 | 0.269 |  |
|  | CT | 186 | 19.47 | 11.33 | 21.05 | 55 | | 22.75 | 8.52 | 24.3 | 0.017 |  |
|  | TT | 77 | 19.36 | 9.09 | 20.5 | 30 | | 28.76 | 33.22 | 25.3 | 0.032 |  |
|  | P-value |  | 0.847 |  |  |  | | 0.450 |  |  |  |  |
|  | CC | 91 | 18.67 | 9.35 | 20.5 | 28 | | 21.21 | 8.27 | 24 | 0.269 |  |
|  | TT + CT | 263 | 19.44 | 10.71 | 20.6 | 85 | | 24.87 | 20.88 | 24.6 | 0.001 |  |
|  | P-value |  | 0.567 |  |  |  | | 0.260 |  |  |  |  |
|  | CC + CT | 277 | 19.21 | 10.71 | 20.7 | 83 | | 22.23 | 8.42 | 24.3 | 0.010 |  |
|  | TT | 77 | 19.36 | 9.09 | 20.5 | 30 | | 28.76 | 33.22 | 25.3 | 0.032 |  |
|  | P-value |  | 0.813 |  |  |  | | 0.356 |  |  |  |  |
|  | Homo | 168 | 18.99 | 9.21 | 20.5 | 58 | | 25.12 | 24.66 | 24.8 | 0.018 |  |
|  | Hetero | 186 | 19.47 | 11.33 | 21.05 | 55 | | 22.75 | 8.52 | 24.3 | 0.017 |  |
|  | P-value |  | 0.760 |  |  |  | | 0.874 |  |  |  |  |

TMPRSS2: Transmembrane Serine Protease 2; CRP: C-Reactive Protein.

Supplementary Table S11: Association TMPRSS2 genotypes distribution with serum CRP level

| Genotypes  Alleles  N (%) | | CRP | | | | | | | | | | |
| --- | --- | --- | --- | --- | --- | --- | --- | --- | --- | --- | --- | --- |
|  |  | Inpatient Intubation -No | | | | | Inpatient Intubation -Yes | | | | P-value |  |
|  |  | N | Mean | SD | Median | N | | Mean | SD | Median |  |  |
| TMPRSS2 rs2070788 | CC | 101 | 18.86 | 9.25 | 20.7 | 18 | | 21.56 | 8.34 | 24 | 0.326 |  |
|  | CT | 204 | 19.86 | 11.14 | 21.55 | 37 | | 22.19 | 8.78 | 23.2 | 0.155 |  |
|  | TT | 89 | 21.18 | 21.13 | 20.5 | 18 | | 26.07 | 5.65 | 27.55 | 0.004 |  |
|  | P-value |  | 0.728 |  |  |  | | 0.149 |  |  |  |  |
|  | CC | 101 | 18.86 | 9.25 | 20.7 | 18 | | 21.56 | 8.34 | 24 | 0.326 |  |
|  | TT + CT | 293 | 20.26 | 14.87 | 21.1 | 55 | | 23.46 | 8.05 | 24.6 | 0.005 |  |
|  | P-value |  | 0.453 |  |  |  | | 0.360 |  |  |  |  |
|  | CC + CT | 305 | 19.53 | 10.55 | 21.2 | 55 | | 21.98 | 8.57 | 23.3 | 0.087 |  |
|  | TT | 89 | 21.18 | 21.13 | 20.5 | 18 | | 26.07 | 5.65 | 27.55 | 0.004 |  |
|  | P-value |  | 0.984 |  |  |  | | 0.054 |  |  |  |  |
|  | Homo | 190 | 19.94 | 15.95 | 20.6 | 36 | | 23.81 | 7.39 | 25.2 | 0.005 |  |
|  | Hetero | 204 | 19.86 | 11.14 | 21.55 | 37 | | 22.19 | 8.78 | 23.2 | 0.155 |  |
|  | P-value |  | 0.502 |  |  |  | | 0.383 |  |  |  |  |

TMPRSS2: Transmembrane Serine Protease 2; CRP: C-Reactive Protein.

Supplementary Table S12: Association TMPRSS2 genotypes distribution with serum CRP level.

| Genotypes  Alleles  N (%) | | CRP | | | | | | | | | | |
| --- | --- | --- | --- | --- | --- | --- | --- | --- | --- | --- | --- | --- |
|  |  | Survived | | | | | Expired | | | | P-value |  |
|  |  | N | Mean | SD | Median | N | | Mean | SD | Median |  |  |
| TMPRSS2 rs2070788 | CC | 161 | 13.88 | 10.63 | 13.4 | 19 | | 21.51 | 8.11 | 23.3 | 0.004 |  |
|  | CT | 314 | 14.8 | 11.98 | 14.85 | 35 | | 23.48 | 8.17 | 24.6 | 0.000 |  |
|  | TT | 149 | 14.41 | 18.6 | 11.3 | 21 | | 24.14 | 7.92 | 27.3 | 0.000 |  |
|  | P-value |  | 0.499 |  |  |  | | 0.426 |  |  |  |  |
|  | CC | 161 | 13.88 | 10.63 | 13.4 | 19 | | 21.51 | 8.11 | 23.3 | 0.004 |  |
|  | TT + CT | 463 | 14.67 | 14.43 | 13.9 | 56 | | 23.73 | 8.01 | 25.25 | 0.000 |  |
|  | P-value |  | 0.712 |  |  |  | | 0.237 |  |  |  |  |
|  | CC + CT | 475 | 14.49 | 11.54 | 14.1 | 54 | | 22.79 | 8.13 | 24.6 | 0.000 |  |
|  | TT | 149 | 14.41 | 18.6 | 11.3 | 21 | | 24.14 | 7.92 | 27.3 | 0.000 |  |
|  | P-value |  | 0.350 |  |  |  | | 0.342 |  |  |  |  |
|  | Homo | 310 | 14.14 | 14.98 | 12.8 | 40 | | 22.89 | 8.02 | 25.05 | 0.000 |  |
|  | Hetero | 314 | 14.8 | 11.98 | 14.85 | 35 | | 23.48 | 8.17 | 24.6 | 0.000 |  |
|  | P-value |  | 0.263 |  |  |  | | 0.861 |  |  |  |  |

TMPRSS2: Transmembrane Serine Protease 2; CRP: C-Reactive Protein.

Supplementary Table S13: Association TMPRSS2 genotypes distribution with serum IL-6 levels.

| Genotypes  Alleles  N (%) | | IL-6 | | | | | | | | | | |
| --- | --- | --- | --- | --- | --- | --- | --- | --- | --- | --- | --- | --- |
|  |  | Control | | | | | COVID-19 | | | | P-value |  |
|  |  | N | Mean | SD | Median | N | | Mean | SD | Median |  |  |
| TMPRSS2 rs2070788 | CC | 12 | 2.94 | 1.47 | 2 | 153 | | 54.24 | 136.28 | 15.6 | 0.000 |  |
|  | CT | 22 | 3.89 | 3.18 | 2 | 268 | | 53.62 | 140.45 | 11.8 | 0.000 |  |
|  | TT | 14 | 5.83 | 5.82 | 2.91 | 123 | | 70.2 | 183.83 | 17 | 0.000 |  |
|  | P-value |  | 0.445 |  |  |  | | 0.113 |  |  |  |  |
|  | CC | 12 | 2.94 | 1.47 | 2 | 153 | | 54.24 | 136.28 | 15.6 | 0.000 |  |
|  | TT + CT | 36 | 4.64 | 4.42 | 2 | 391 | | 58.84 | 155.35 | 13.5 | 0.000 |  |
|  | P-value |  | 0.396 |  |  |  | | 0.620 |  |  |  |  |
|  | CC + CT | 34 | 3.55 | 2.71 | 2 | 421 | | 53.85 | 138.79 | 13.1 | 0.000 |  |
|  | TT | 14 | 5.83 | 5.82 | 2.91 | 123 | | 70.2 | 183.83 | 17 | 0.000 |  |
|  | P-value |  | 0.232 |  |  |  | | 0.081 |  |  |  |  |
|  | Homo | 26 | 4.5 | 4.55 | 2 | 276 | | 61.35 | 159.13 | 16.35 | 0.000 |  |
|  | Hetero | 22 | 3.89 | 3.18 | 2 | 268 | | 53.62 | 140.45 | 11.8 | 0.000 |  |
|  | P-value |  | 0.725 |  |  |  | | 0.057 |  |  |  |  |

TMPRSS2: Transmembrane Serine Protease 2; IL: interleukin.

Supplementary Table S14: Association TMPRSS2 genotypes distribution with serum IL-6 levels.

| Genotypes  Alleles  N (%) | | IL-6 | | | | | | | | | | |
| --- | --- | --- | --- | --- | --- | --- | --- | --- | --- | --- | --- | --- |
|  |  | Outpatient | | | | | Inpatient | | | | P-value |  |
|  |  | N | Mean | SD | Median | N | | Mean | SD | Median |  |  |
| TMPRSS2 rs2070788 | CC | 48 | 10.41 | 13.52 | 4.57 | 105 | | 74.28 | 160.53 | 24.6 | 0.000 |  |
|  | CT | 71 | 14.67 | 29.65 | 3.43 | 197 | | 67.66 | 160.65 | 15.6 | 0.000 |  |
|  | TT | 34 | 77.25 | 239.25 | 4.27 | 89 | | 67.5 | 159.25 | 29.1 | 0.000 |  |
|  | P-value |  | 0.444 |  |  |  | | 0.056 |  |  |  |  |
|  | CC | 48 | 10.41 | 13.52 | 4.57 | 105 | | 74.28 | 160.53 | 24.6 | 0.000 |  |
|  | TT + CT | 105 | 34.94 | 140.07 | 3.51 | 286 | | 67.61 | 159.94 | 17.55 | 0.000 |  |
|  | P-value |  | 0.920 |  |  |  | | 0.130 |  |  |  |  |
|  | CC + CT | 119 | 12.95 | 24.47 | 3.67 | 302 | | 69.96 | 160.37 | 19.4 | 0.000 |  |
|  | TT | 34 | 77.25 | 239.25 | 4.27 | 89 | | 67.5 | 159.25 | 29.1 | 0.000 |  |
|  | P-value |  | 0.222 |  |  |  | | 0.207 |  |  |  |  |
|  | Homo | 82 | 38.12 | 156.6 | 4.47 | 194 | | 71.17 | 159.57 | 25.94 | 0.000 |  |
|  | Hetero | 71 | 14.67 | 29.65 | 3.43 | 197 | | 67.66 | 160.65 | 15.6 | 0.000 |  |
|  | P-value |  | 0.355 |  |  |  | | 0.016 |  |  |  |  |

TMPRSS2: Transmembrane Serine Protease 2; IL: interleukin.

Supplementary Table S15: Association TMPRSS2 genotypes distribution with serum IL-6 levels. Non-Severe: Outpatients + Inpatients (not ICU)

Severe: ICU _+_ Intubated

| Genotypes  Alleles  N (%) | | IL-6 | | | | | | | | | | |
| --- | --- | --- | --- | --- | --- | --- | --- | --- | --- | --- | --- | --- |
|  |  | Non-Severe | | | | | Severe | | | | P-value |  |
|  |  | N | Mean | SD | Median | N | | Mean | SD | Median |  |  |
| TMPRSS2 rs2070788 | CC | 125 | 29.3 | 45.26 | 13.6 | 28 | | 165.61 | 281.8 | 37.15 | 0.001 |  |
|  | CT | 222 | 40.93 | 113.29 | 10.05 | 46 | | 114.89 | 222.2 | 23 | 0.000 |  |
|  | TT | 95 | 63.36 | 176.63 | 15.3 | 28 | | 93.37 | 208.24 | 30.8 | 0.077 |  |
|  | P-value |  | 0.156 |  |  |  | | 0.581 |  |  |  |  |
|  | CC | 125 | 29.3 | 45.26 | 13.6 | 28 | | 165.61 | 281.8 | 37.15 | 0.001 |  |
|  | TT + CT | 317 | 47.65 | 135.51 | 11.9 | 74 | | 106.75 | 215.83 | 27.05 | 0.000 |  |
|  | P-value |  | 0.826 |  |  |  | | 0.299 |  |  |  |  |
|  | CC + CT | 347 | 36.74 | 94.67 | 10.6 | 74 | | 134.08 | 245.8 | 29.7 | 0.000 |  |
|  | TT | 95 | 63.36 | 176.63 | 15.3 | 28 | | 93.37 | 208.24 | 30.8 | 0.077 |  |
|  | P-value |  | 0.083 |  |  |  | | 0.642 |  |  |  |  |
|  | Homo | 220 | 44.01 | 121.81 | 14.45 | 56 | | 129.49 | 248.19 | 31.35 | 0.000 |  |
|  | Hetero | 222 | 40.93 | 113.29 | 10.05 | 46 | | 114.89 | 222.2 | 23 | 0.000 |  |
|  | P-value |  | 0.104 |  |  |  | | 0.607 |  |  |  |  |

TMPRSS2: Transmembrane Serine Protease 2; IL: interleukin.

Supplementary Table S16: Association TMPRSS2 genotypes distribution with serum IL-6 levels.

| Genotypes  Alleles  N (%) | | IL-6 | | | | | | | | | | |
| --- | --- | --- | --- | --- | --- | --- | --- | --- | --- | --- | --- | --- |
|  |  | Inpatient ICU-No | | | | | Inpatient ICU-Yes | | | | P-value |  |
|  |  | N | Mean | SD | Median | N | | Mean | SD | Median |  |  |
| TMPRSS2 rs2070788 | CC | 77 | 41.07 | 53.51 | 23.5 | 28 | | 165.61 | 281.8 | 37.15 | 0.076 |  |
|  | CT | 151 | 53.28 | 134.24 | 14.9 | 46 | | 114.89 | 222.2 | 23 | 0.018 |  |
|  | TT | 61 | 55.62 | 131.24 | 24.1 | 28 | | 93.37 | 208.24 | 30.8 | 0.707 |  |
|  | P-value |  | 0.060 |  |  |  | | 0.581 |  |  |  |  |
|  | CC | 77 | 41.07 | 53.51 | 23.5 | 28 | | 165.61 | 281.8 | 37.15 | 0.076 |  |
|  | TT + CT | 212 | 53.95 | 133.07 | 16.05 | 74 | | 106.75 | 215.83 | 27.05 | 0.019 |  |
|  | P-value |  | 0.222 |  |  |  | | 0.299 |  |  |  |  |
|  | CC + CT | 228 | 49.15 | 113.57 | 17.25 | 74 | | 134.08 | 245.8 | 29.7 | 0.002 |  |
|  | TT | 61 | 55.62 | 131.24 | 24.1 | 28 | | 93.37 | 208.24 | 30.8 | 0.707 |  |
|  | P-value |  | 0.120 |  |  |  | | 0.642 |  |  |  |  |
|  | Homo | 138 | 47.5 | 95.84 | 23.75 | 56 | | 129.49 | 248.19 | 31.35 | 0.115 |  |
|  | Hetero | 151 | 53.28 | 134.24 | 14.9 | 46 | | 114.89 | 222.2 | 23 | 0.018 |  |
|  | P-value |  | 0.019 |  |  |  | | 0.607 |  |  |  |  |

TMPRSS2: Transmembrane Serine Protease 2; IL: interleukin.

Supplementary Table S17: Association TMPRSS2 genotypes distribution with serum IL-6 levels.

| Genotypes  Alleles  N (%) | | IL-6 | | | | | | | | | | |
| --- | --- | --- | --- | --- | --- | --- | --- | --- | --- | --- | --- | --- |
|  |  | Inpatient Intubation -No | | | | | Inpatient Intubation -Yes | | | | P-value |  |
|  |  | N | Mean | SD | Median | N | | Mean | SD | Median |  |  |
| TMPRSS2 rs2070788 | CC | 85 | 42.25 | 54.46 | 23.5 | 20 | | 210.4 | 322.26 | 37.95 | 0.058 |  |
|  | CT | 163 | 50.83 | 129.71 | 14 | 34 | | 148.34 | 250.17 | 55.8 | 0.001 |  |
|  | TT | 70 | 53.56 | 126.36 | 19 | 19 | | 118.85 | 243.38 | 38.1 | 0.033 |  |
|  | P-value |  | 0.052 |  |  |  | | 0.955 |  |  |  |  |
|  | CC | 85 | 42.25 | 54.46 | 23.5 | 20 | | 210.4 | 322.26 | 37.95 | 0.058 |  |
|  | TT + CT | 233 | 51.65 | 128.45 | 15.3 | 53 | | 137.77 | 245.82 | 45.1 | 0.000 |  |
|  | P-value |  | 0.092 |  |  |  | | 0.771 |  |  |  |  |
|  | CC + CT | 248 | 47.89 | 109.82 | 16.6 | 54 | | 171.33 | 277.7 | 48.8 | 0.000 |  |
|  | TT | 70 | 53.56 | 126.36 | 19 | 19 | | 118.85 | 243.38 | 38.1 | 0.033 |  |
|  | P-value |  | 0.263 |  |  |  | | 0.980 |  |  |  |  |
|  | Homo | 155 | 47.36 | 93.83 | 21.2 | 39 | | 165.8 | 286.59 | 38.1 | 0.005 |  |
|  | Hetero | 163 | 50.83 | 129.71 | 14 | 34 | | 148.34 | 250.17 | 55.8 | 0.001 |  |
|  | P-value |  | 0.016 |  |  |  | | 0.812 |  |  |  |  |

TMPRSS2: Transmembrane Serine Protease 2; IL: interleukin.

Supplementary Table S18: Association TMPRSS2 genotypes distribution with serum IL-6 levels.

| Genotypes  Alleles  N (%) | | IL-6 | | | | | | | | | | |
| --- | --- | --- | --- | --- | --- | --- | --- | --- | --- | --- | --- | --- |
|  |  | Survived | | | | | Expired | | | | P-value |  |
|  |  | N | Mean | SD | Median | N | | Mean | SD | Median |  |  |
| TMPRSS2 rs2070788 | CC | 131 | 29.24 | 46.09 | 13.8 | 22 | | 203.12 | 306.87 | 59.9 | 0.000 |  |
|  | CT | 239 | 46.6 | 129.46 | 10.4 | 29 | | 111.51 | 204.3 | 45.1 | 0.000 |  |
|  | TT | 103 | 61.09 | 171.53 | 14.9 | 20 | | 117.11 | 237.23 | 40.6 | 0.002 |  |
|  | P-value |  | 0.247 |  |  |  | | 0.654 |  |  |  |  |
|  | CC | 131 | 29.24 | 46.09 | 13.8 | 22 | | 203.12 | 306.87 | 59.9 | 0.000 |  |
|  | TT + CT | 342 | 50.96 | 143.33 | 11.75 | 49 | | 113.8 | 215.94 | 43.1 | 0.000 |  |
|  | P-value |  | 0.823 |  |  |  | | 0.361 |  |  |  |  |
|  | CC + CT | 370 | 40.45 | 107.83 | 10.9 | 51 | | 151.03 | 254.99 | 45.1 | 0.000 |  |
|  | TT | 103 | 61.09 | 171.53 | 14.9 | 20 | | 117.11 | 237.23 | 40.6 | 0.002 |  |
|  | P-value |  | 0.135 |  |  |  | | 0.783 |  |  |  |  |
|  | Homo | 234 | 43.26 | 119.65 | 14.3 | 42 | | 162.16 | 276.05 | 42.3 | 0.000 |  |
|  | Hetero | 239 | 46.6 | 129.46 | 10.4 | 29 | | 111.51 | 204.3 | 45.1 | 0.000 |  |
|  | P-value |  | 0.152 |  |  |  | | 0.543 |  |  |  |  |

TMPRSS2: Transmembrane Serine Protease 2; IL: interleukin.

Supplementary Table S19: Association TMPRSS2 genotypes distribution with serum IL-1β level.

| Genotypes  Alleles  N (%) | | IL-1β | | | | | | | | | | |
| --- | --- | --- | --- | --- | --- | --- | --- | --- | --- | --- | --- | --- |
|  |  | Control | | | | | COVID-19 | | | | P-value |  |
|  |  | N | Mean | SD | Median | N | | Mean | SD | Median |  |  |
| TMPRSS2 rs2070788 | CC | 14 | 12.94 | 30.67 | 3.9 | 168 | | 13.28 | 42.51 | 3.9 | 0.281 |  |
|  | CT | 29 | 8.57 | 18.87 | 3.9 | 312 | | 12.16 | 37.14 | 3.9 | 0.867 |  |
|  | TT | 16 | 6.06 | 4.46 | 3.9 | 147 | | 12.7 | 39.21 | 3.9 | 0.027 |  |
|  | P-value |  | 0.270 |  |  |  | | 0.162 |  |  |  |  |
|  | CC | 14 | 12.94 | 30.67 | 3.9 | 168 | | 13.28 | 42.51 | 3.9 | 0.281 |  |
|  | TT + CT | 45 | 7.68 | 15.32 | 3.9 | 459 | | 12.34 | 37.78 | 3.9 | 0.263 |  |
|  | P-value |  | 0.400 |  |  |  | | 0.111 |  |  |  |  |
|  | CC + CT | 43 | 9.99 | 23.09 | 3.9 | 480 | | 12.56 | 39.06 | 3.9 | 0.639 |  |
|  | TT | 16 | 6.06 | 4.46 | 3.9 | 147 | | 12.7 | 39.21 | 3.9 | 0.027 |  |
|  | P-value |  | 0.311 |  |  |  | | 0.129 |  |  |  |  |
|  | Homo | 30 | 9.27 | 21.08 | 3.9 | 315 | | 13.01 | 40.94 | 3.9 | 0.028 |  |
|  | Hetero | 29 | 8.57 | 18.87 | 3.9 | 312 | | 12.16 | 37.14 | 3.9 | 0.867 |  |
|  | P-value |  | 0.106 |  |  |  | | 0.899 |  |  |  |  |

TMPRSS2: Transmembrane Serine Protease 2; IL: interleukin.

Supplementary Table S20: Association TMPRSS2 genotypes distribution with serum IL-1β level.

| Genotypes  Alleles  N (%) | | IL-1β | | | | | | | | | | |
| --- | --- | --- | --- | --- | --- | --- | --- | --- | --- | --- | --- | --- |
|  |  | Outpatient | | | | | Inpatient | | | | P-value |  |
|  |  | N | Mean | SD | Median | N | | Mean | SD | Median |  |  |
| TMPRSS2 rs2070788 | CC | 55 | 19.12 | 56.47 | 3.9 | 113 | | 10.44 | 33.64 | 3.9 | 0.749 |  |
|  | CT | 103 | 18.35 | 49.96 | 3.9 | 209 | | 9.12 | 28.47 | 3.9 | 0.004 |  |
|  | TT | 58 | 15.83 | 43.48 | 3.9 | 89 | | 10.67 | 36.27 | 3.9 | 0.184 |  |
|  | P-value |  | 0.531 |  |  |  | | 0.061 |  |  |  |  |
|  | CC | 55 | 19.12 | 56.47 | 3.9 | 113 | | 10.44 | 33.64 | 3.9 | 0.749 |  |
|  | TT + CT | 161 | 17.44 | 47.6 | 3.9 | 298 | | 9.58 | 30.95 | 3.9 | 0.002 |  |
|  | P-value |  | 0.911 |  |  |  | | 0.022 |  |  |  |  |
|  | CC + CT | 158 | 18.62 | 52.14 | 3.9 | 322 | | 9.58 | 30.34 | 3.9 | 0.015 |  |
|  | TT | 58 | 15.83 | 43.48 | 3.9 | 89 | | 10.67 | 36.27 | 3.9 | 0.184 |  |
|  | P-value |  | 0.314 |  |  |  | | 0.183 |  |  |  |  |
|  | Homo | 113 | 17.43 | 50.02 | 3.9 | 202 | | 10.54 | 34.74 | 3.9 | 0.353 |  |
|  | Hetero | 103 | 18.35 | 49.96 | 3.9 | 209 | | 9.12 | 28.47 | 3.9 | 0.004 |  |
|  | P-value |  | 0.322 |  |  |  | | 0.347 |  |  |  |  |

TMPRSS2: Transmembrane Serine Protease 2; IL: interleukin.

Supplementary Table S21: Association TMPRSS2 genotypes distribution with serum IL-1β level.

Non-Severe: Outpatients + Inpatients (not ICU)

Severe: ICU _+_ Intubated

| Genotypes  Alleles  N (%) | | IL-1β | | | | | | | | | | |
| --- | --- | --- | --- | --- | --- | --- | --- | --- | --- | --- | --- | --- |
|  |  | Non-Severe | | | | | Severe | | | | P-value |  |
|  |  | N | Mean | SD | Median | N | | Mean | SD | Median |  |  |
| TMPRSS2 rs2070788 | CC | 140 | 14.2 | 45.95 | 3.9 | 28 | | 8.69 | 16.78 | 3.9 | 0.341 |  |
|  | CT | 266 | 13.13 | 39.73 | 3.9 | 46 | | 6.57 | 14.23 | 3.9 | 0.270 |  |
|  | TT | 124 | 12.6 | 38.56 | 3.9 | 23 | | 13.27 | 43.47 | 3.9 | 0.847 |  |
|  | P-value |  | 0.370 |  |  |  | | 0.339 |  |  |  |  |
|  | CC | 140 | 14.2 | 45.95 | 3.9 | 28 | | 8.69 | 16.78 | 3.9 | 0.341 |  |
|  | TT + CT | 390 | 12.96 | 39.31 | 3.9 | 69 | | 8.8 | 27.48 | 3.9 | 0.412 |  |
|  | P-value |  | 0.214 |  |  |  | | 0.316 |  |  |  |  |
|  | CC + CT | 406 | 13.5 | 41.92 | 3.9 | 74 | | 7.37 | 15.17 | 3.9 | 0.136 |  |
|  | TT | 124 | 12.6 | 38.56 | 3.9 | 23 | | 13.27 | 43.47 | 3.9 | 0.847 |  |
|  | P-value |  | 0.299 |  |  |  | | 0.174 |  |  |  |  |
|  | Homo | 264 | 13.45 | 42.56 | 3.9 | 51 | | 10.76 | 31.44 | 3.9 | 0.492 |  |
|  | Hetero | 266 | 13.13 | 39.73 | 3.9 | 46 | | 6.57 | 14.23 | 3.9 | 0.270 |  |
|  | P-value |  | 0.828 |  |  |  | | 0.804 |  |  |  |  |

TMPRSS2: Transmembrane Serine Protease 2; IL: interleukin.

Supplementary Table S22: Association TMPRSS2 genotypes distribution with serum IL-1β level.

| Genotypes  Alleles  N (%) | | IL-1β | | | | | | | | | | |
| --- | --- | --- | --- | --- | --- | --- | --- | --- | --- | --- | --- | --- |
|  |  | Inpatient ICU-No | | | | | Inpatient ICU-Yes | | | | P-value |  |
|  |  | N | Mean | SD | Median | N | | Mean | SD | Median |  |  |
| TMPRSS2 rs2070788 | CC | 85 | 11.02 | 37.65 | 3.9 | 28 | | 8.69 | 16.78 | 3.9 | 0.252 |  |
|  | CT | 163 | 9.84 | 31.34 | 3.9 | 46 | | 6.57 | 14.23 | 3.9 | 0.018 |  |
|  | TT | 66 | 9.76 | 33.74 | 3.9 | 23 | | 13.27 | 43.47 | 3.9 | 0.758 |  |
|  | P-value |  | 0.128 |  |  |  | | 0.339 |  |  |  |  |
|  | CC | 85 | 11.02 | 37.65 | 3.9 | 28 | | 8.69 | 16.78 | 3.9 | 0.252 |  |
|  | TT + CT | 229 | 9.81 | 31.98 | 3.9 | 69 | | 8.8 | 27.48 | 3.9 | 0.033 |  |
|  | P-value |  | 0.043 |  |  |  | | 0.316 |  |  |  |  |
|  | CC + CT | 248 | 10.24 | 33.57 | 3.9 | 74 | | 7.37 | 15.17 | 3.9 | 0.010 |  |
|  | TT | 66 | 9.76 | 33.74 | 3.9 | 23 | | 13.27 | 43.47 | 3.9 | 0.758 |  |
|  | P-value |  | 0.462 |  |  |  | | 0.174 |  |  |  |  |
|  | Homo | 151 | 10.47 | 35.88 | 3.9 | 51 | | 10.76 | 31.44 | 3.9 | 0.273 |  |
|  | Hetero | 163 | 9.84 | 31.34 | 3.9 | 46 | | 6.57 | 14.23 | 3.9 | 0.018 |  |
|  | P-value |  | 0.230 |  |  |  | | 0.804 |  |  |  |  |

TMPRSS2: Transmembrane Serine Protease 2; IL: interleukin.

Supplementary Table S23: Association TMPRSS2 genotypes distribution with serum IL-1β level.

| Genotypes  Alleles  N (%) | | IL-1β | | | | | | | | | | |
| --- | --- | --- | --- | --- | --- | --- | --- | --- | --- | --- | --- | --- |
|  |  | Inpatient Intubation -No | | | | | Inpatient Intubation -Yes | | | | P-value |  |
|  |  | N | Mean | SD | Median | N | | Mean | SD | Median |  |  |
| TMPRSS2 rs2070788 | CC | 95 | 10.34 | 35.65 | 3.9 | 18 | | 11.01 | 20.75 | 3.9 | 0.602 |  |
|  | CT | 176 | 9.43 | 30.19 | 3.9 | 33 | | 7.42 | 16.77 | 3.9 | 0.088 |  |
|  | TT | 72 | 12.17 | 40.22 | 3.9 | 17 | | 4.31 | 1.65 | 3.9 | 1.000 |  |
|  | P-value |  | 0.079 |  |  |  | | 0.464 |  |  |  |  |
|  | CC | 95 | 10.34 | 35.65 | 3.9 | 18 | | 11.01 | 20.75 | 3.9 | 0.602 |  |
|  | TT + CT | 248 | 10.23 | 33.35 | 3.9 | 50 | | 6.36 | 13.66 | 3.9 | 0.164 |  |
|  | P-value |  | 0.025 |  |  |  | | 0.503 |  |  |  |  |
|  | CC + CT | 271 | 9.75 | 32.15 | 3.9 | 51 | | 8.69 | 18.15 | 3.9 | 0.107 |  |
|  | TT | 72 | 12.17 | 40.22 | 3.9 | 17 | | 4.31 | 1.65 | 3.9 | 1.000 |  |
|  | P-value |  | 0.355 |  |  |  | | 0.226 |  |  |  |  |
|  | Homo | 167 | 11.13 | 37.58 | 3.9 | 35 | | 7.75 | 15.1 | 3.9 | 0.777 |  |
|  | Hetero | 176 | 9.43 | 30.19 | 3.9 | 33 | | 7.42 | 16.77 | 3.9 | 0.088 |  |
|  | P-value |  | 0.210 |  |  |  | | 0.648 |  |  |  |  |

TMPRSS2: Transmembrane Serine Protease 2; IL: interleukin.

Supplementary Table S24: Association TMPRSS2 genotypes distribution with serum IL-1β level.

| Genotypes  Alleles  N (%) | | IL-1β | | | | | | | | | | |
| --- | --- | --- | --- | --- | --- | --- | --- | --- | --- | --- | --- | --- |
|  |  | Survived | | | | | Expired | | | | P-value |  |
|  |  | N | Mean | SD | Median | N | | Mean | SD | Median |  |  |
| TMPRSS2 rs2070788 | CC | 149 | 13.61 | 44.59 | 3.9 | 19 | | 10.69 | 20.21 | 3.9 | 0.426 |  |
|  | CT | 281 | 12.63 | 38.71 | 3.9 | 31 | | 7.95 | 17.31 | 3.9 | 0.160 |  |
|  | TT | 131 | 13.73 | 41.43 | 3.9 | 16 | | 4.33 | 1.7 | 3.9 | 0.759 |  |
|  | P-value |  | 0.289 |  |  |  | | 0.356 |  |  |  |  |
|  | CC | 149 | 13.61 | 44.59 | 3.9 | 19 | | 10.69 | 20.21 | 3.9 | 0.426 |  |
|  | TT + CT | 412 | 12.98 | 39.55 | 3.9 | 47 | | 6.72 | 14.12 | 3.9 | 0.324 |  |
|  | P-value |  | 0.153 |  |  |  | | 0.525 |  |  |  |  |
|  | CC + CT | 430 | 12.97 | 40.79 | 3.9 | 50 | | 8.99 | 18.32 | 3.9 | 0.104 |  |
|  | TT | 131 | 13.73 | 41.43 | 3.9 | 16 | | 4.33 | 1.7 | 3.9 | 0.759 |  |
|  | P-value |  | 0.272 |  |  |  | | 0.153 |  |  |  |  |
|  | Homo | 280 | 13.67 | 43.06 | 3.9 | 35 | | 7.79 | 15.1 | 3.9 | 0.662 |  |
|  | Hetero | 281 | 12.63 | 38.71 | 3.9 | 31 | | 7.95 | 17.31 | 3.9 | 0.160 |  |
|  | P-value |  | 0.738 |  |  |  | | 0.514 |  |  |  |  |

TMPRSS2: Transmembrane Serine Protease 2; IL: interleukin.

Supplementary Table S25: Association TMPRSS2 genotypes distribution with serum TNF-α level.

| Genotypes  Alleles  N (%) | | TNF-α | | | | | | | | | | |
| --- | --- | --- | --- | --- | --- | --- | --- | --- | --- | --- | --- | --- |
|  |  | Control | | | | | COVID-19 | | | | P-value |  |
|  |  | N | Mean | SD | Median | N | | Mean | SD | Median |  |  |
| TMPRSS2 rs2070788 | CC | 13 | 27.66 | 22.55 | 15.6 | 168 | | 66.75 | 199.45 | 15.6 | 0.573 |  |
|  | CT | 29 | 28.81 | 46.78 | 15.6 | 311 | | 45 | 135.21 | 15.6 | 0.992 |  |
|  | TT | 16 | 39.47 | 35.94 | 24.12 | 147 | | 41.6 | 122.72 | 15.6 | 0.008 |  |
|  | P-value |  | 0.145 |  |  |  | | 0.499 |  |  |  |  |
|  | CC | 13 | 27.66 | 22.55 | 15.6 | 168 | | 66.75 | 199.45 | 15.6 | 0.573 |  |
|  | TT + CT | 45 | 32.6 | 43.12 | 15.6 | 458 | | 43.91 | 131.21 | 15.6 | 0.102 |  |
|  | P-value |  | 0.908 |  |  |  | | 0.505 |  |  |  |  |
|  | CC + CT | 42 | 28.45 | 40.54 | 15.6 | 479 | | 52.63 | 160.82 | 15.6 | 0.754 |  |
|  | TT | 16 | 39.47 | 35.94 | 24.12 | 147 | | 41.6 | 122.72 | 15.6 | 0.008 |  |
|  | P-value |  | 0.059 |  |  |  | | 0.254 |  |  |  |  |
|  | Homo | 29 | 34.18 | 30.75 | 15.6 | 315 | | 55.01 | 168.28 | 15.6 | 0.019 |  |
|  | Hetero | 29 | 28.81 | 46.78 | 15.6 | 311 | | 45 | 135.21 | 15.6 | 0.992 |  |
|  | P-value |  | 0.111 |  |  |  | | 0.707 |  |  |  |  |

Supplementary Table S26: Association TMPRSS2 genotypes distribution with serum TNF-α level.

| Genotypes  Alleles  N (%) | | TNF-α | | | | | | | | | | |
| --- | --- | --- | --- | --- | --- | --- | --- | --- | --- | --- | --- | --- |
|  |  | Outpatient | | | | | Inpatient | | | | P-value |  |
|  |  | N | Mean | SD | Median | N | | Mean | SD | Median |  |  |
| TMPRSS2 rs2070788 | CC | 55 | 92.02 | 242.35 | 15.6 | 113 | | 54.44 | 174.74 | 15.6 | 0.221 |  |
|  | CT | 103 | 74.02 | 198.9 | 15.6 | 208 | | 30.63 | 85.2 | 15.6 | 0.071 |  |
|  | TT | 58 | 54.68 | 145.82 | 15.6 | 89 | | 33.08 | 105.02 | 15.6 | 0.627 |  |
|  | P-value |  | 0.203 |  |  |  | | 0.947 |  |  |  |  |
|  | CC | 55 | 92.02 | 242.35 | 15.6 | 113 | | 54.44 | 174.74 | 15.6 | 0.221 |  |
|  | TT + CT | 161 | 67.06 | 181.33 | 15.6 | 297 | | 31.36 | 91.41 | 15.6 | 0.255 |  |
|  | P-value |  | 0.470 |  |  |  | | 0.746 |  |  |  |  |
|  | CC + CT | 158 | 80.29 | 214.42 | 15.6 | 321 | | 39.01 | 124.54 | 15.6 | 0.033 |  |
|  | TT | 58 | 54.68 | 145.82 | 15.6 | 89 | | 33.08 | 105.02 | 15.6 | 0.627 |  |
|  | P-value |  | 0.074 |  |  |  | | 0.956 |  |  |  |  |
|  | Homo | 113 | 72.86 | 198.72 | 15.6 | 202 | | 45.03 | 148.17 | 15.6 | 0.648 |  |
|  | Hetero | 103 | 74.02 | 198.9 | 15.6 | 208 | | 30.63 | 85.2 | 15.6 | 0.071 |  |
|  | P-value |  | 0.341 |  |  |  | | 0.807 |  |  |  |  |

TMPRSS2: Transmembrane Serine Protease 2; TNF: tumor necrosis factor.

Supplementary Table S27: Association TMPRSS2 genotypes distribution with serum TNF-α level.

Non-Severe: Outpatients + Inpatients (not ICU)

Severe: ICU _+_ Intubated

| Genotypes  Alleles  N (%) | | TNF-α | | | | | | | | | | |
| --- | --- | --- | --- | --- | --- | --- | --- | --- | --- | --- | --- | --- |
|  |  | Non-Severe | | | | | Severe | | | | P-value |  |
|  |  | N | Mean | SD | Median | N | | Mean | SD | Median |  |  |
| TMPRSS2 rs2070788 | CC | 140 | 69.58 | 202.59 | 15.6 | 28 | | 52.56 | 185.75 | 15.6 | 0.172 |  |
|  | CT | 265 | 47.89 | 145.33 | 15.6 | 46 | | 28.37 | 41.22 | 15.6 | 0.496 |  |
|  | TT | 124 | 44.33 | 133.25 | 15.6 | 23 | | 26.91 | 20.45 | 15.6 | 0.471 |  |
|  | P-value |  | 0.241 |  |  |  | | 0.619 |  |  |  |  |
|  | CC | 140 | 69.58 | 202.59 | 15.6 | 28 | | 52.56 | 185.75 | 15.6 | 0.172 |  |
|  | TT + CT | 389 | 46.75 | 141.43 | 15.6 | 69 | | 27.88 | 35.5 | 15.6 | 0.882 |  |
|  | P-value |  | 0.286 |  |  |  | | 0.440 |  |  |  |  |
|  | CC + CT | 405 | 55.39 | 167.42 | 15.6 | 74 | | 37.52 | 118.1 | 15.6 | 0.172 |  |
|  | TT | 124 | 44.33 | 133.25 | 15.6 | 23 | | 26.91 | 20.45 | 15.6 | 0.471 |  |
|  | P-value |  | 0.113 |  |  |  | | 0.402 |  |  |  |  |
|  | Homo | 264 | 57.72 | 173.65 | 15.6 | 51 | | 40.99 | 137.78 | 15.6 | 0.616 |  |
|  | Hetero | 265 | 47.89 | 145.33 | 15.6 | 46 | | 28.37 | 41.22 | 15.6 | 0.496 |  |
|  | P-value |  | 0.688 |  |  |  | | 0.989 |  |  |  |  |

TMPRSS2: Transmembrane Serine Protease 2; TNF: tumor necrosis factor.

Supplementary Table S28: Association TMPRSS2 genotypes distribution with serum TNF-α level.

| Genotypes  Alleles  N (%) | | TNF-α | | | | | | | | | | |
| --- | --- | --- | --- | --- | --- | --- | --- | --- | --- | --- | --- | --- |
|  |  | Inpatient ICU-No | | | | | Inpatient ICU-Yes | | | | P-value |  |
|  |  | N | Mean | SD | Median | N | | Mean | SD | Median |  |  |
| TMPRSS2 rs2070788 | CC | 85 | 55.07 | 172.1 | 15.6 | 28 | | 52.56 | 185.75 | 15.6 | 0.276 |  |
|  | CT | 162 | 31.27 | 94.1 | 15.6 | 46 | | 28.37 | 41.22 | 15.6 | 0.861 |  |
|  | TT | 66 | 35.23 | 121.54 | 15.6 | 23 | | 26.91 | 20.45 | 15.6 | 0.508 |  |
|  | P-value |  | 0.677 |  |  |  | | 0.619 |  |  |  |  |
|  | CC | 85 | 55.07 | 172.1 | 15.6 | 28 | | 52.56 | 185.75 | 15.6 | 0.276 |  |
|  | TT + CT | 228 | 32.42 | 102.54 | 15.6 | 69 | | 27.88 | 35.5 | 15.6 | 0.807 |  |
|  | P-value |  | 0.408 |  |  |  | | 0.440 |  |  |  |  |
|  | CC + CT | 247 | 39.46 | 126.64 | 15.6 | 74 | | 37.52 | 118.1 | 15.6 | 0.415 |  |
|  | TT | 66 | 35.23 | 121.54 | 15.6 | 23 | | 26.91 | 20.45 | 15.6 | 0.508 |  |
|  | P-value |  | 0.579 |  |  |  | | 0.402 |  |  |  |  |
|  | Homo | 151 | 46.39 | 151.94 | 15.6 | 51 | | 40.99 | 137.78 | 15.6 | 0.724 |  |
|  | Hetero | 162 | 31.27 | 94.1 | 15.6 | 46 | | 28.37 | 41.22 | 15.6 | 0.861 |  |
|  | P-value |  | 0.777 |  |  |  | | 0.989 |  |  |  |  |

TMPRSS2: Transmembrane Serine Protease 2; TNF: tumor necrosis factor.

Supplementary Table S29: Association TMPRSS2 genotypes distribution with serum TNF-α level.

| Genotypes  Alleles  N (%) | | TNF-α | | | | | | | | | | |
| --- | --- | --- | --- | --- | --- | --- | --- | --- | --- | --- | --- | --- |
|  |  | Inpatient Intubation -No | | | | | Inpatient Intubation -Yes | | | | P-value |  |
|  |  | N | Mean | SD | Median | N | | Mean | SD | Median |  |  |
| TMPRSS2 rs2070788 | CC | 95 | 51.18 | 163.1 | 15.6 | 18 | | 71.65 | 231.72 | 15.6 | 0.459 |  |
|  | CT | 175 | 30.11 | 90.61 | 15.6 | 33 | | 33.39 | 47.93 | 15.6 | 0.181 |  |
|  | TT | 72 | 34.68 | 116.44 | 15.6 | 17 | | 26.28 | 20.95 | 15.6 | 0.664 |  |
|  | P-value |  | 0.692 |  |  |  | | 0.580 |  |  |  |  |
|  | CC | 95 | 51.18 | 163.1 | 15.6 | 18 | | 71.65 | 231.72 | 15.6 | 0.459 |  |
|  | TT + CT | 247 | 31.44 | 98.61 | 15.6 | 50 | | 30.98 | 40.68 | 15.6 | 0.183 |  |
|  | P-value |  | 0.400 |  |  |  | | 0.320 |  |  |  |  |
|  | CC + CT | 270 | 37.52 | 121.28 | 15.6 | 51 | | 46.9 | 141.66 | 15.6 | 0.542 |  |
|  | TT | 72 | 34.68 | 116.44 | 15.6 | 17 | | 26.28 | 20.95 | 15.6 | 0.664 |  |
|  | P-value |  | 0.911 |  |  |  | | 0.965 |  |  |  |  |
|  | Homo | 167 | 44.07 | 144.67 | 15.6 | 35 | | 49.62 | 166.08 | 15.6 | 0.808 |  |
|  | Hetero | 175 | 30.11 | 90.61 | 15.6 | 33 | | 33.39 | 47.93 | 15.6 | 0.181 |  |
|  | P-value |  | 0.507 |  |  |  | | 0.401 |  |  |  |  |

TMPRSS2: Transmembrane Serine Protease 2; TNF: tumor necrosis factor.

Supplementary Table S30: Association TMPRSS2 genotypes distribution with serum TNF-α level.

| Genotypes  Alleles  N (%) | | TNF-α | | | | | | | | | | |
| --- | --- | --- | --- | --- | --- | --- | --- | --- | --- | --- | --- | --- |
|  |  | Survived | | | | | Expired | | | | P-value |  |
|  |  | N | Mean | SD | Median | N | | Mean | SD | Median |  |  |
| TMPRSS2 rs2070788 | CC | 149 | 66.48 | 196.73 | 15.6 | 19 | | 68.86 | 225.52 | 15.6 | 0.476 |  |
|  | CT | 280 | 44.81 | 136.92 | 15.6 | 31 | | 46.76 | 120.64 | 15.6 | 0.836 |  |
|  | TT | 131 | 43.68 | 129.72 | 15.6 | 16 | | 24.62 | 20.44 | 15.6 | 0.974 |  |
|  | P-value |  | 0.456 |  |  |  | | 0.955 |  |  |  |  |
|  | CC | 149 | 66.48 | 196.73 | 15.6 | 19 | | 68.86 | 225.52 | 15.6 | 0.476 |  |
|  | TT + CT | 411 | 44.45 | 134.51 | 15.6 | 47 | | 39.22 | 98.69 | 15.6 | 0.877 |  |
|  | P-value |  | 0.426 |  |  |  | | 0.814 |  |  |  |  |
|  | CC + CT | 429 | 52.33 | 160.35 | 15.6 | 50 | | 55.15 | 166.47 | 15.6 | 0.561 |  |
|  | TT | 131 | 43.68 | 129.72 | 15.6 | 16 | | 24.62 | 20.44 | 15.6 | 0.974 |  |
|  | P-value |  | 0.239 |  |  |  | | 0.924 |  |  |  |  |
|  | Homo | 280 | 55.81 | 168.82 | 15.6 | 35 | | 48.63 | 166.16 | 15.6 | 0.633 |  |
|  | Hetero | 280 | 44.81 | 136.92 | 15.6 | 31 | | 46.76 | 120.64 | 15.6 | 0.836 |  |
|  | P-value |  | 0.769 |  |  |  | | 0.768 |  |  |  |  |

TMPRSS2: Transmembrane Serine Protease 2; TNF: tumor necrosis factor.
